# Inverse design of anisotropic bone scaffold based on machine learning and regenerative genetic algorithm

**DOI:** 10.3389/fbioe.2023.1241151

**Published:** 2023-09-07

**Authors:** Wenhang Liu, Youwei Zhang, Yongtao Lyu, Sergei Bosiakov, Yadong Liu

**Affiliations:** ^1^ Department of Engineering Mechanics, Dalian University of Technology, Dalian, China; ^2^ DUT-BSU Joint Institute, Dalian University of Technology, Dalian, China; ^3^ Faculty of Mechanics and Mathematics, Belarusian State University, Minsk, Belarus; ^4^ Department of Orthopedics, Dalian Municipal Central Hospital Affiliated of Dalian University of Technology, Dalian, China

**Keywords:** machine learning, genetic algorithm, triply periodic minimal surfaces, inverse design, arrangement anisotropy

## Abstract

**Introduction:** Triply periodic minimal surface (TPMS) is widely used in the design of bone scaffolds due to its structural advantages. However, the current approach to designing bone scaffolds using TPMS structures is limited to a forward process from microstructure to mechanical properties. Developing an inverse bone scaffold design method based on the mechanical properties of bone structures is crucial.

**Methods:** Using the machine learning and genetic algorithm, a new inverse design model was proposed in this research. The anisotropy of bone was matched by changing the number of cells in different directions. The finite element (FE) method was used to calculate the TPMS configuration and generate a back propagation neural network (BPNN) data set. Neural networks were used to establish the relationship between microstructural parameters and the elastic matrix of bone. This relationship was then used with regenerative genetic algorithm (RGA) in inverse design.

**Results:** The accuracy of the BPNN-RGA model was confirmed by comparing the elasticity matrix of the inverse-designed structure with that of the actual bone. The results indicated that the average error was below 3.00% for three mechanical performance parameters as design targets, and approximately 5.00% for six design targets.

**Discussion:** The present study demonstrated the potential of combining machine learning with traditional optimization method to inversely design anisotropic TPMS bone scaffolds with target mechanical properties. The BPNN-RGA model achieves higher design efficiency, compared to traditional optimization methods. The entire design process is easily controlled.

## 1 Introduction

Bone is a crucial part of the human body, serving various functions such as body support, protection of internal organs, and mineral storage. With the increasing aging population, the number of people suffering from joint diseases is also rising, leading to a greater demand for external repair techniques for bone defects (Gruskin et al., 2012; Li et al., 2015; Lin et al., 2020). Currently, the most important treatment method for repairing bone defects is bone tissue engineering scaffolds that can guide bone tissue regeneration (Henkel et al., 2013; Tang et al., 2016; Zhu et al., 2021). The triply periodic minimal surface (TPMS) is an ideal model for designing scaffolds in bone tissue engineering due to its zero mean curvature and high specific surface area, which is similar to natural bone (Yan et al., 2015; Bobbert et al., 2017). However, it should be noted that different parts of bone tissue have varying mechanical properties, and the mechanical properties of the same bone tissue may differ in different directions. Therefore, it is crucial to develop an inverse design method for bone scaffolds based on the mechanical properties of bone structures.

The current research on TPMS bone scaffold mainly focuses on optimizing its structure to achieve the target performance. For example, (Yánez et al., 2018), systematically investigated stress conditions under compression and torsion of different types of Gyroid porous structures with varying porosity models (Rajagopalan and Robb, 2006). Proposed two bracket models, the P-type bracket and the regular voxel bracket in the TPMS unit. They found that the stress distribution of the P-unit bracket was better than that of other units with smaller strains (Wieding et al., 2014). Optimized the configuration parameters of titanium alloy scaffolds with opening characteristics, making the scaffolds have similarly elastic to human bone and satisfactory pore size. Overall, the research on TPMS bone scaffolds still needs to be improved, focusing on optimizing their structures to improve their performance. Unlike the previous uniform arrangement, the anisotropic TPMS structure is introduced in this article. This structure is more in line with the real structure of bone, which is also anisotropic.

To realize the inverse design, the machine learning (ML) based method is resorted due to its low computational cost, high adaptability to various physical problems, and good independence from physical models. ML-based method is a data-driven method, and its effectiveness depends on the amount of prepared data and the algorithm employed (Wang et al., 2021). Besides, the use of ML for inverse design has matured in metamaterials. For example, the artificial neural network (ANN) was employed by (Peurifoy et al., 2018) to approximate the inverse design of photonic crystals. The deep-learning-based model comprising two bidirectional ANN was established to design and optimize the chiroptical metamaterials at specific wavelengths (Ma et al., 2018). Recently, the Gauss-Bayesian model involving Bayesian optimization using Gaussian kernel was proposed to realize the inverse design of various acoustic metamaterials for predesignated functionality (Zheng et al., 2020). While ML methods have been effectively utilized in the inverse design of metamaterials, their application in bone implants is still limited and requires further investigation.

**FIGURE 1:**
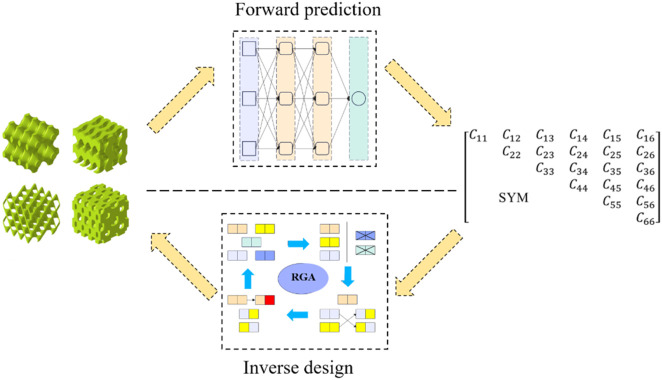
Flow chart of forward prediction and inverse design.

The present study aimed to inversely design complex bone scaffolds using anisotropic TPMS structures. The target for inverse design was the partial elasticity matrix of bone. A mapping relationship between structural parameters and mechanical properties using the back propagation neural network (BPNN) neural network was established in our design method. Then, a regenerative genetic algorithm (RGA) was embedded in machine learning for inverse search to obtain the desired structure (Figure 1). Finally, several sets of design targets and high-precision finite element (FE) simulations were used to demonstrate the validity and generalizability of the BPNN-RGA model.

## 2 Materials and methods

### 2.1 Anisotropic TPMS structures

**FIGURE 2:**
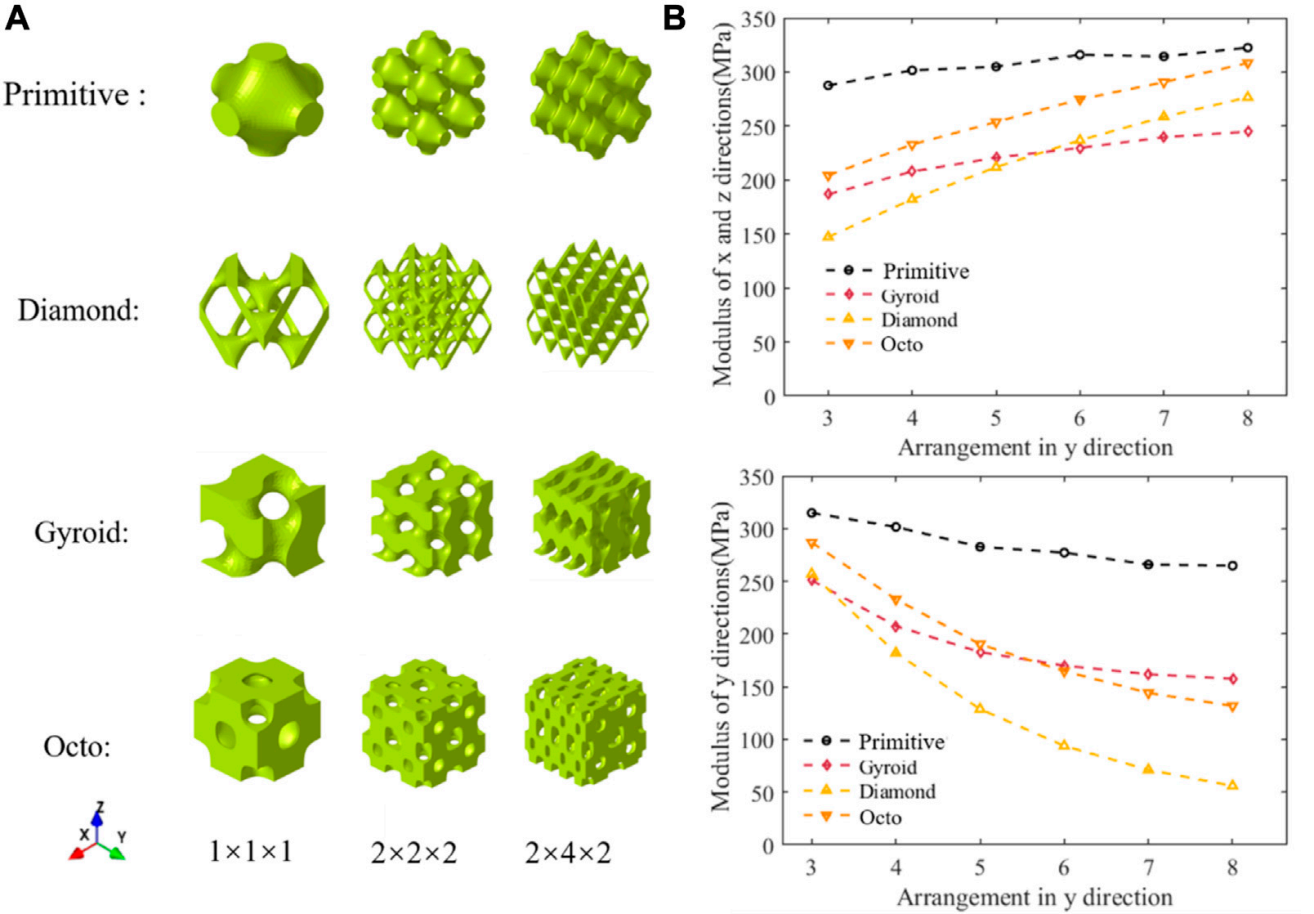
Schematic diagram of structure and their performance. **(A)** Structures of various arrangements **(B)** Variation of compression modulus with the number of arrangements.

Arrangement anisotropy refers to the fact that the number of TPMS unit cells arranged in each direction is different while the length of the structure in each direction remains constant. The whole structure had different mechanical properties, such as Young’s modulus, in different directions. As shown in Figure 2A, the unit cell structure, normal arrangement structure, and anisotropic arrangement structure of four types of TPMS required for inverse design are displayed. As shown in Figure 2B, the change in compressive modulus in different directions of the structure varies with the number of units in the y -direction of the given coordinate system in Figure 2A. It should be noted that when only the modulus in the y -direction is changed, the moduli in the x -direction and z -direction were the same (the number in *x* and *z* directions is kept “2”). A more complicated structural design can be made if the numbers in the three directions are different.

Anisotropic TPMS structures can be generated by controlling the parameters of the surface equations. The anisotropic Primitive scaffold was characterized using the following equation:

$$U_{S}=\cos\left(\frac{2\pi m}{L}x\right)+\cos\left(\frac{2\pi n}{L}y\right)+\cos\left(\frac{2\pi l}{L}z\right)-t$$

where m , n , and l are the arrangement numbers of the anisotropic Primitive unit cell in the x , y , and z directions, respectively; t is the surface control coefficient, which is related to porosity φ ; L is the dimension of the design structure in the x , y , and z directions.

**FIGURE 3:**
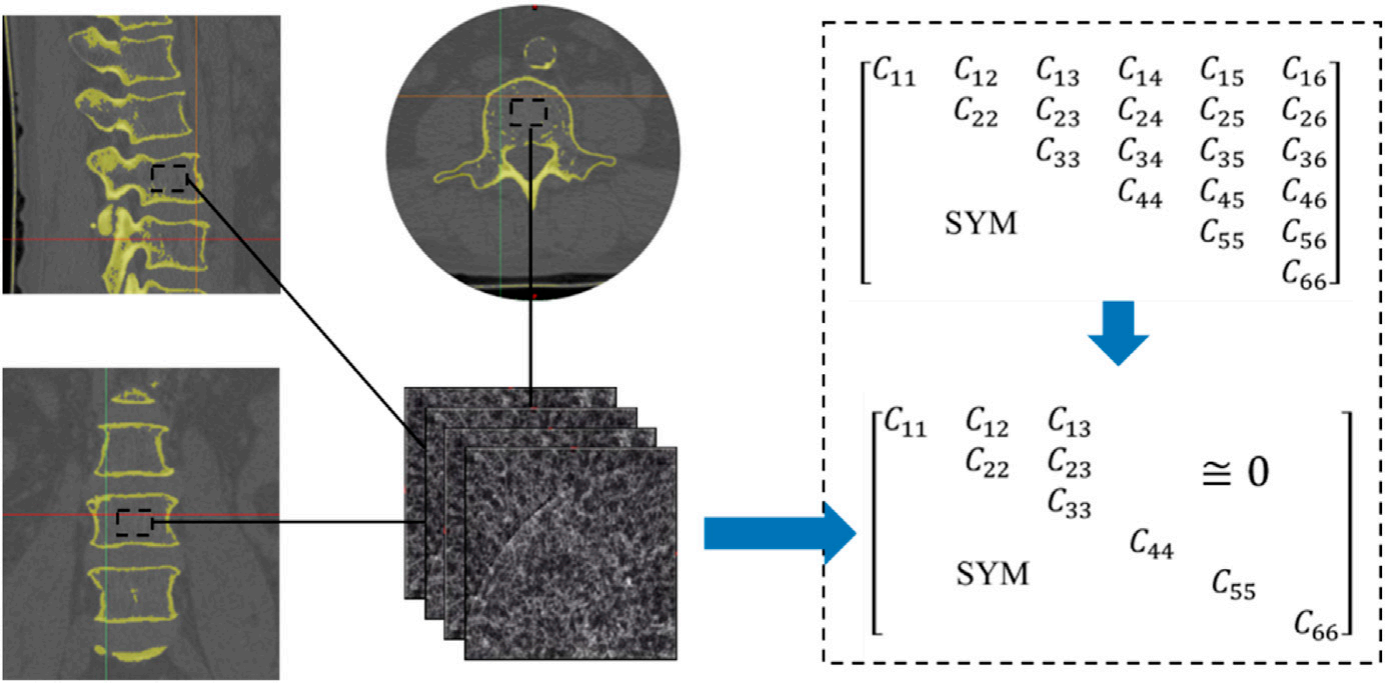
CT images of bone structure and its stiffness matrix.

The FE method was used to calculate the equivalent stiffness matrix of the human bone from computed tomography (CT) images (Figure 3). The 2D image is re-established as a 3D model by superposition, and then the finite element method is utilized to solve the $C_{ij}$ , with 1,2,3 in the parameters corresponding to the *x*, *y*, and *z* directions, respectively, as detailed in the literature (Xiao et al., 2021; Lu et al., 2022). The stiffness matrix was then compared with those in the existing literature to verify its rationality (Kalouche et al., 2010; Wang et al., 2016). The analysis of the equivalent elastic matrix data shows that it is difficult to achieve inverse design of the elastic matrix if the TPMS was uniformly arranged in space. A previous study has shown that, in a specific direction, the mechanical properties of TPMS scaffolds can be significantly improved by adjusting structural anisotropy (Peng et al., 2022). This feature was used to create structures with various mechanical properties in all directions.

### 2.2 Establishment of machine learning database

The ML method is largely database dependent, so enough data are required to perform the inverse design. To generate the data set for ML to predict the equivalent stiffness matrix of TPMS, we obtained the 3D scatter plot from the TPMS surface equation and used the scatter plot to generate the unit node information. The range of variation of the number of structural arrangements is set to 3–8. The range of variation of porosity is set to 50%–75%. The element node information was imported into ANSYS (v.18.0, Ansys Inc., Canonsburg, PA, United States) to generate the FE model. Moreover, FE calculations were performed according to different boundary conditions. TPMS surface parameters and the results of FE calculation were used as the training set of neural networks.

**FIGURE 4:**
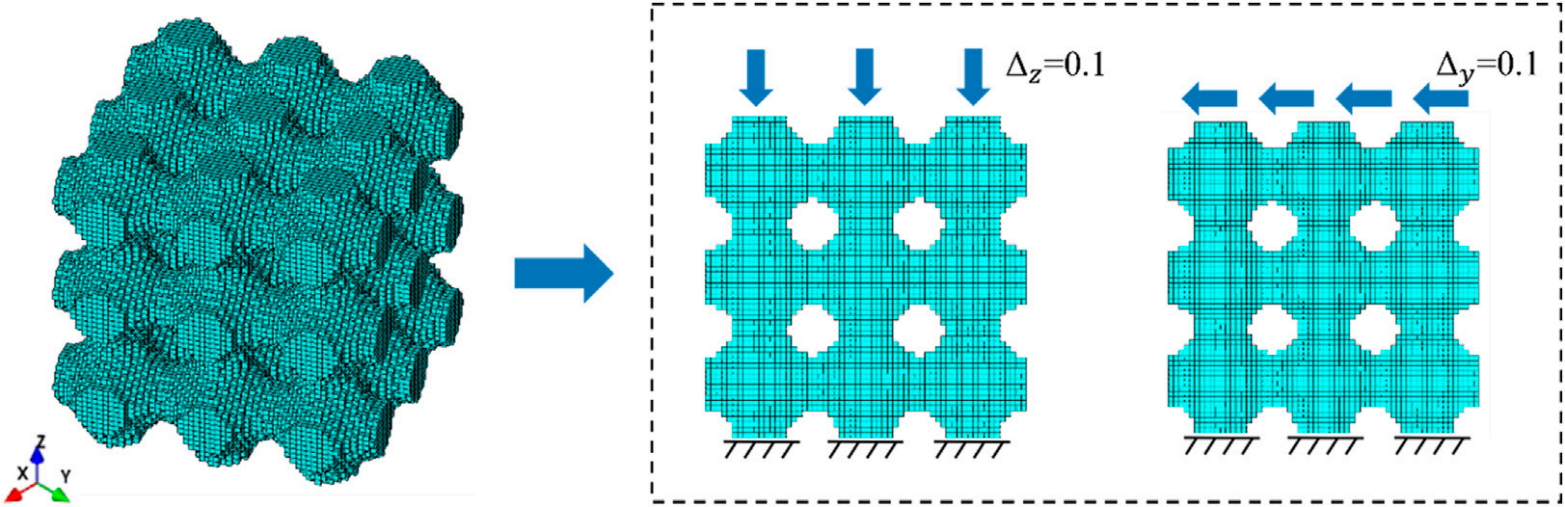
Two loading conditions of the TPMS structure.

As shown in the Figure 4, the compression and shear terms in the stiffness matrix were solved using the unidirectional compression and pure shear conditions. The lower surface of the TPMS structure was completely fixed and a displacement load of 0.1 was applied to the upper edge face. The length L was 10.00 mm, and the elements were first-order hexahedral solid elements (Solid185). The Young’s modulus for the component material was 10.00 GPa, and the Poisson ratio was 0.30 (Hak et al., 2014; Wu et al., 2018). It should be noted that Young’s modulus of the designed configuration was changed to 100 GPa for the low porosity bone due to the significant difference between the two bone moduli (Collins et al., 2021).

### 2.3 Forward prediction using BPNN

**FIGURE 5:**
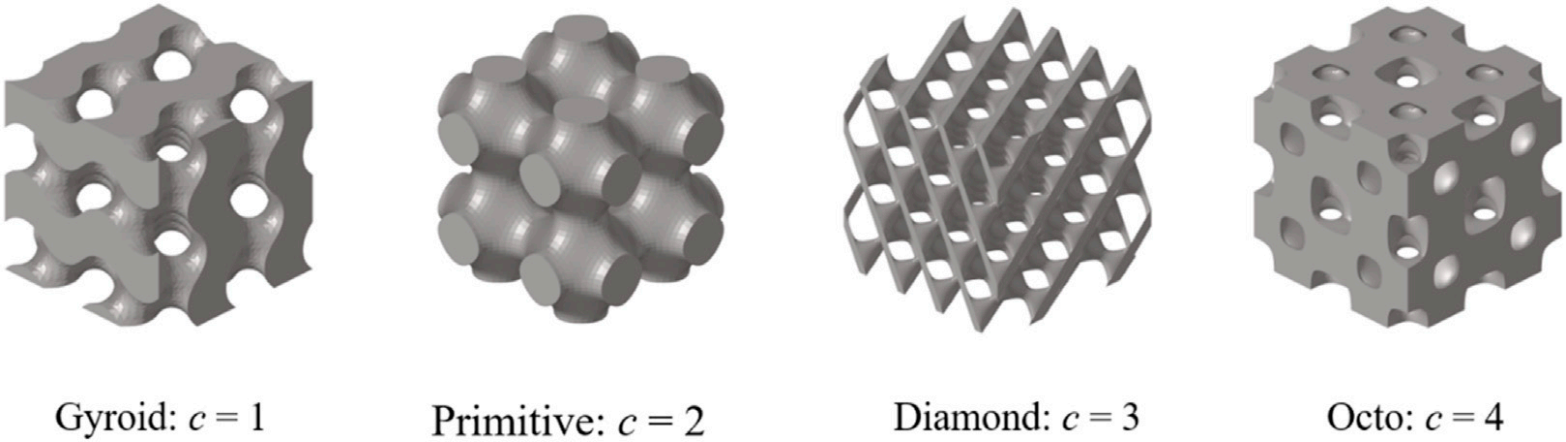
Machine learning label of TPMS structures.

ML can be used to quickly predict problems that are previously difficult to solve (Yan et al., 2018; Chen et al., 2019). The BPNN is a representative ML algorithm inspired by the biological neural network of the human brain (Lu et al., 2019). BPNN could be regarded as a non-linear operator, which takes an input vector *X* and returns the hypothesis value of the output vector y , as given in equation:

$$y=BPNN\left(X\right)=BPNN\left(c,\varphi ,t,m,n,l\right)$$

where c is a label used to distinguish different TPMS structures, and c is an integer, the value of which is between 1 and 4 (Figure 5).

**FIGURE 6:**
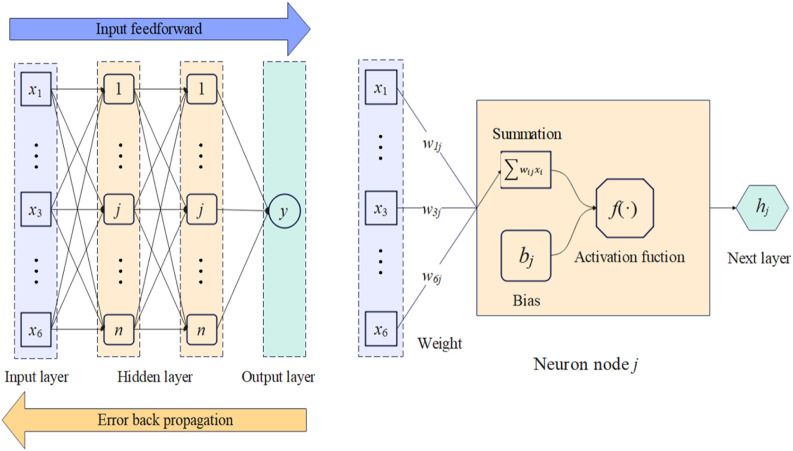
The structure of the BPNN neural network.

A typical BPNN is shown in Figure 6. The first layer of the BPNN was the input layer, the last layer was the output layer, and two hidden layers were introduced between them. When the input information X was transferred into a neuron node in the hidden layer, as shown in Figure 6, the neuron node would give an approximation adjusted by a nonlinear activation function. The nonlinear relationship between the input variables and the medium approximation $h_{j}$ was captured in hidden layer 1.

$$h_{j}=f\left[\left(\sum_{i=1}^{6}w_{ij}^{\left(1\right)}x_{i}\right)+b_{j}^{\left(1\right)}\right],j=1\to m$$

where $w_{ij}^{\left(1\right)}$ is the weight connecting the input variable i and the neuron node j , $b_{j}^{\left(1\right)}$ is the related bias, and f is the nonlinear activation function which is continuous and differentiable.

Through two hidden layers, this approximation is mapped into the output variable ${\tilde{y}}_{j}$ corresponding to the neuron node j by a linear transfer function ϕ .

$$\tilde{y}=\sum_{j=1}^{6}{\tilde{y}}_{j}=\sum_{j=1}^{6}\phi \left(w_{j}^{\left(3\right)}h_{j}+b^{\left(3\right)}\right)$$

where $w_{j}^{\left(3\right)}$ is the weight connecting the neuron j and the output variable $b^{\left(3\right)}$ is the related bias. If the actual output $\tilde{y}$ is different to the target output y , a back propagation of error based on gradient error theory is required to iteratively adjust the weight coefficients in the network to minimize the difference through the mean square error (MSE) function.

Two neural networks, BPNN1 and BPNN2, were trained after determining the optimal structure of the neural network. The BPNN1 was used to train the mapping relationship between the structure and the compressive modulus, and BPNN2 was used to train the mapping relationship between the structure and the shear modulus.

### 2.4 Inverse design using BPNN-RGA

**FIGURE 7:**
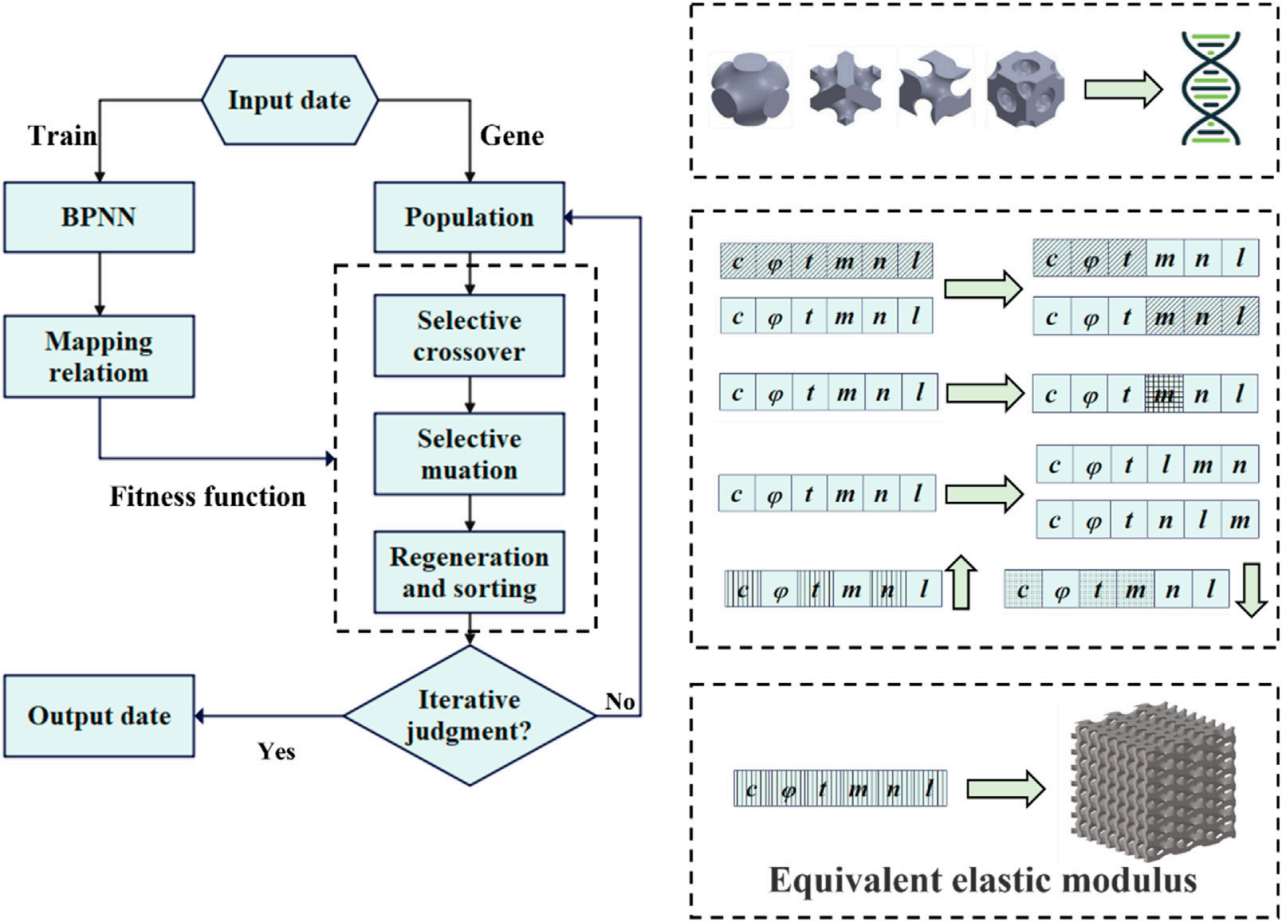
Flow chart of the inverse search using genetic algorithm.

The RGA was employed to search for the TPMS configuration, of which the equivalent stiffness matrix was closest to the objective one, as illustrated in Figure 7. The individuals of the RGA were TPMS configurations, and chromosomes were surface equation parameters. A ML model was used to map the relationship between the surface parameters and the equivalent stiffness. The model’s fitness was determined by comparing the absolute value of the difference between the fitting stiffness and the target stiffness. The smaller the absolute value, the better the fitness. A single output was used to ensure the neural network’s accuracy. Moreover, a regeneration step based on the traditional genetic algorithm was incorporated in this article. Regenerating two structures with the same design parameters but different x , y , and z arrangements was involved in this step. This step aimed to change the direction of load application and obtain the compression or shear properties of the same structure in different directions. Without adding new neural networks, the accuracy of multi-output neural networks was improved in this approach.

TPMS structures with different porosities, arrangement numbers, and cell types were generated as the initial population. The first step in the genetic algorithm was to evaluate the fitness of the individuals in the population. Individuals with lower fitness scores were more likely to undergo cross-mutation, while those with higher fitness scores may also undergo cross-mutation but with a lower probability. After completing the mutations and crossovers, the parents and children were sorted together. The top 2,000 individuals with the highest fitness were selected as the new population, and the next round of mutation and crossover sorting was carried out. The best-adapted individual was recorded each time, and if the error did not decrease after 20 consecutive iterations, it was considered that the best individual had been found. At this point, the outputs were the structural design parameters.

## 3 Result

### 3.1 Optimization of the neural network model

**FIGURE 8:**
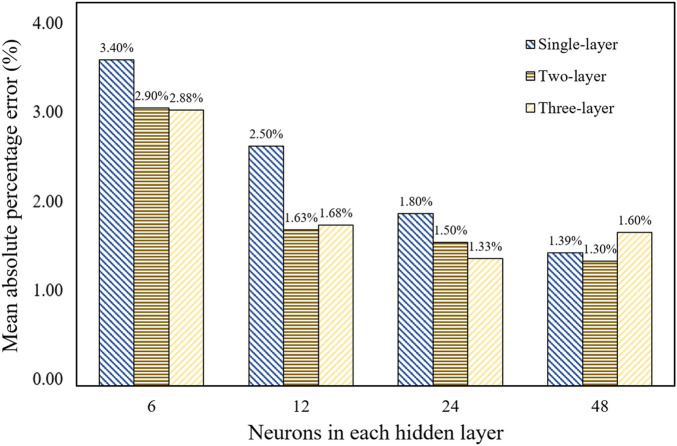
Comparison of the neural network errors with different structures.

We generated 8,000 TPMS configurations and computed their equivalent stiffness matrixes using the FE method, among which 7,000 configurations were used in the training set and 1,000 configurations in the test set. Once the database was established, it was crucial to determine the optimal BPNN structure, including the number of hidden layers and neurons in each layer. The quality of the neural network structure was evaluated based on the mean absolute percentage error (MAPE). The MAPE for the stiffness of each architecture is shown in Figure 8. It can be seen that the BPNN architecture with two hidden layers and 48 neurons in each hidden layer has the minimal error. Therefore, the neural network structure was used to predict the modulus.

**FIGURE 9:**
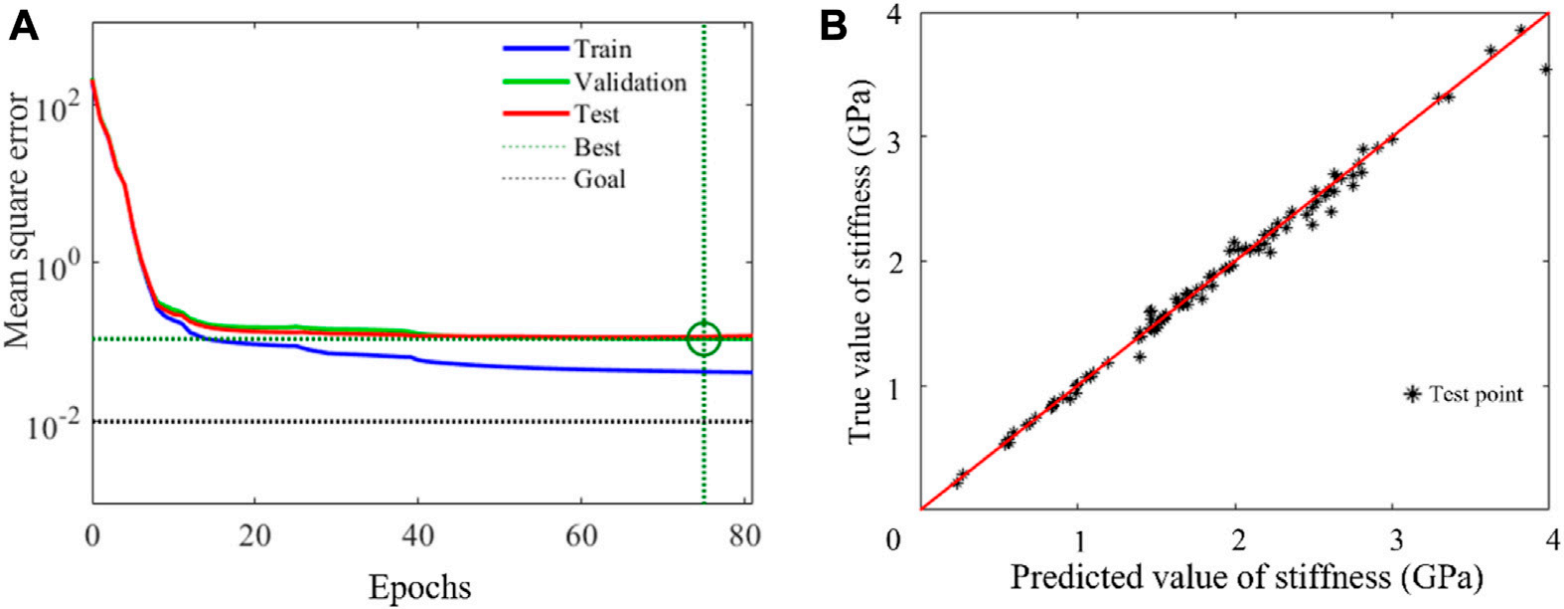
Error analysis of neural network model. **(A)** The mean square error **(B)** The test results of the BPNN network.

The convergent behavior of the selected BPNN architecture is shown in Figure 9A. It was also indicated that there was no overfitting phenomenon because the established BPNN model performed well with training and test datasets. In order to verify the reliability of the neural network, the trained network was loaded, the newly generated input data was given, and the comparison between the fitted output data and that of the FE calculation was made. The result is shown in Figure 9B. It can be seen that the BPNN has a high predictive accuracy even when it is used to predict untrained data. A sensitivity analysis of the number of trainings was also conducted and the results are shown in Table 1. The time consumed to compute the samples was also labeled.

**TABLE 1:** Data sensitivity analysis.

Number of data	Data calculation time (h)	Network training time (s)	Percentage relative error (%)
400	0.36	3	3.5
1,200	1.1	8	2.4
2,000	1.8	8	1.8
4,000	3.6	26	1.4
8,000	7.2	34	1.2

### 3.2 Inverse design based on the porous bone

The stiffness matrix of porous bone was calculated from CT images. However, due to the anisotropy of porous bone, there were still nine different design goals, even after omitting items close to zero. The inverse design of the spine bone focused on the structure’s performance in compression rather than shear. Therefore, the compressive moduli in three directions ( $C_{11},C_{22},C_{33}$ ) were considered as the primary design target, while the shear moduli related to ( $C_{44},C_{55},C_{66}$ ) were the secondary design target. The other non-diagonal items related to compression ( $C_{12},C_{13},C_{23}$ ) were used as the verification items, and their errors should not be too large. Using the regenerative genetic algorithm in inverse design, the two neural networks BPNN1 and BPNN2, combined with the function of the regenerative genetic algorithm, we can obtain the structural design parameters that meet the six design objectives:

$$X_{best}=\left(c,\varphi ,t,m,n,l\right)=\left(1,67\%,0.52,7,8,3\right)$$

**FIGURE 10:**
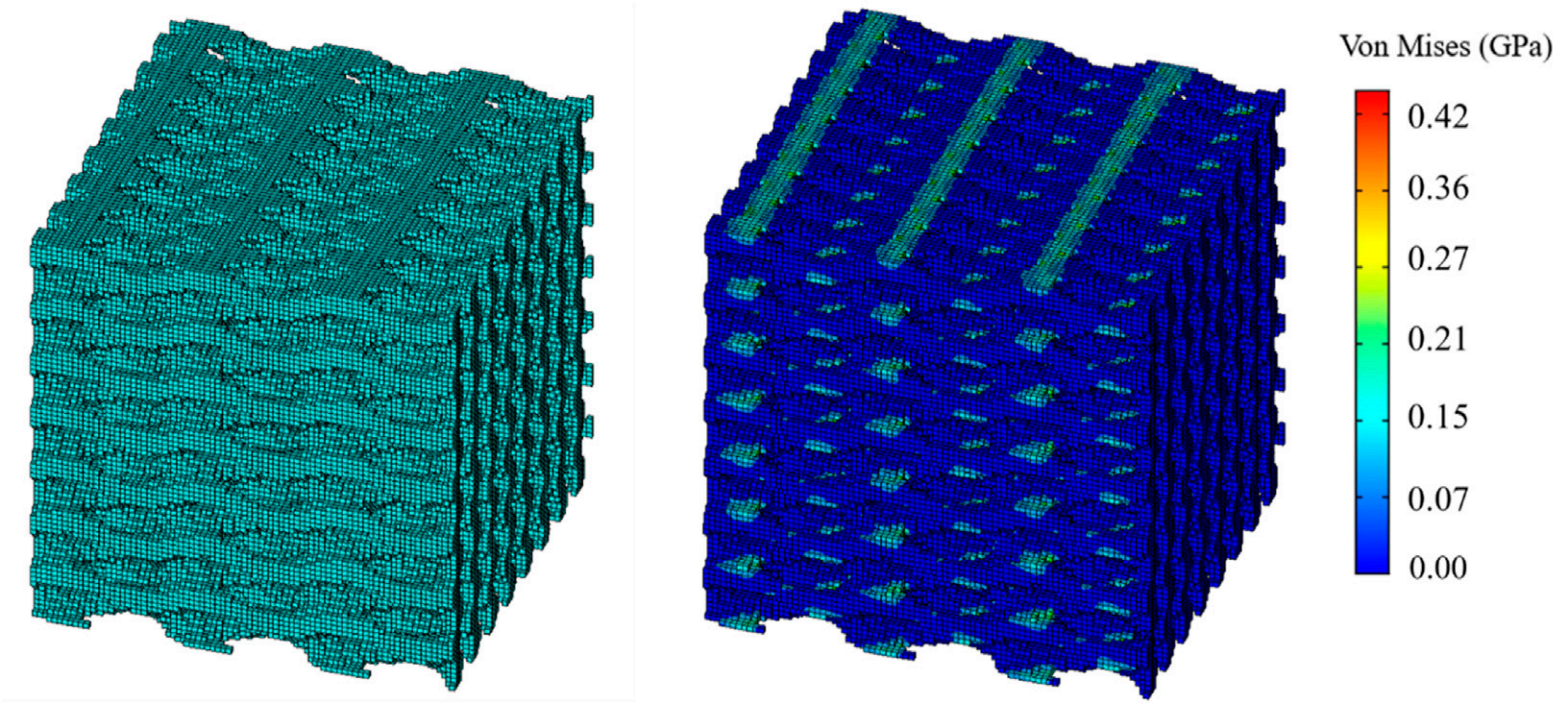
Verification of the design structure using the FE simulation.

**FIGURE 11:**
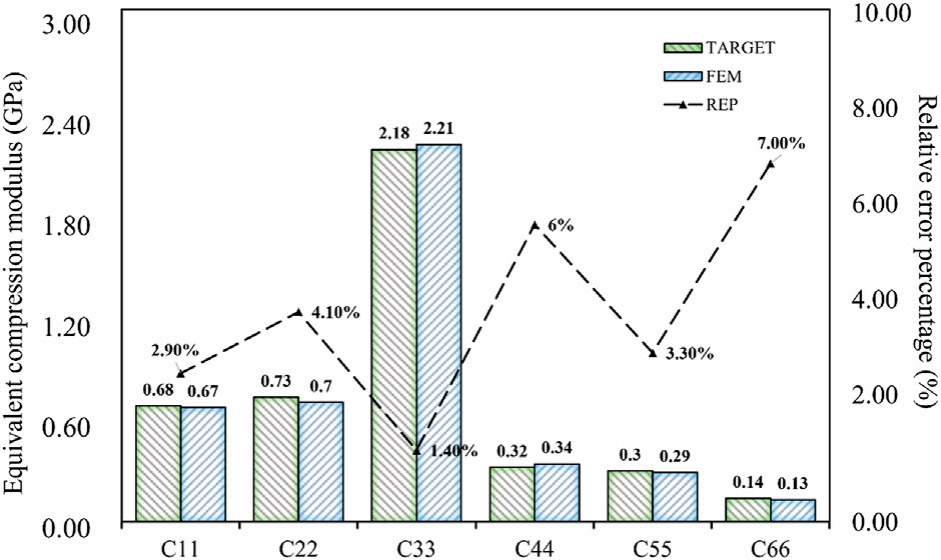
Error analysis of six design objectives of porous bone.

The corresponding TPMS structure was Gyroid, the structural porosity was 67%, and the numbers in the x , y , and z directions were 7, 8, and 3, respectively. The stress distribution under compression is shown in Figure 10. The proposed supporting reaction force can be obtained through formula calculation (Feng et al., 2021; Lu et al., 2022), and the partial stiffness matrix of the structure was compared with the target value. The comparison between the results and the target value is as shown in Figure 11. Error analysis in terms of both numerical magnitude and relative error percentage (REP).

The results of the simulation demonstrated that the BPNN-RGA method could achieve the inverse design with a maximum absolute error of 0.03 GPa and a maximum relative error percentage of 7.00%, when the design targets were the compression and the shear modulus in three directions. The secondary design target had the most significant error percentage due to its small magnitude.

**FIGURE 12:**
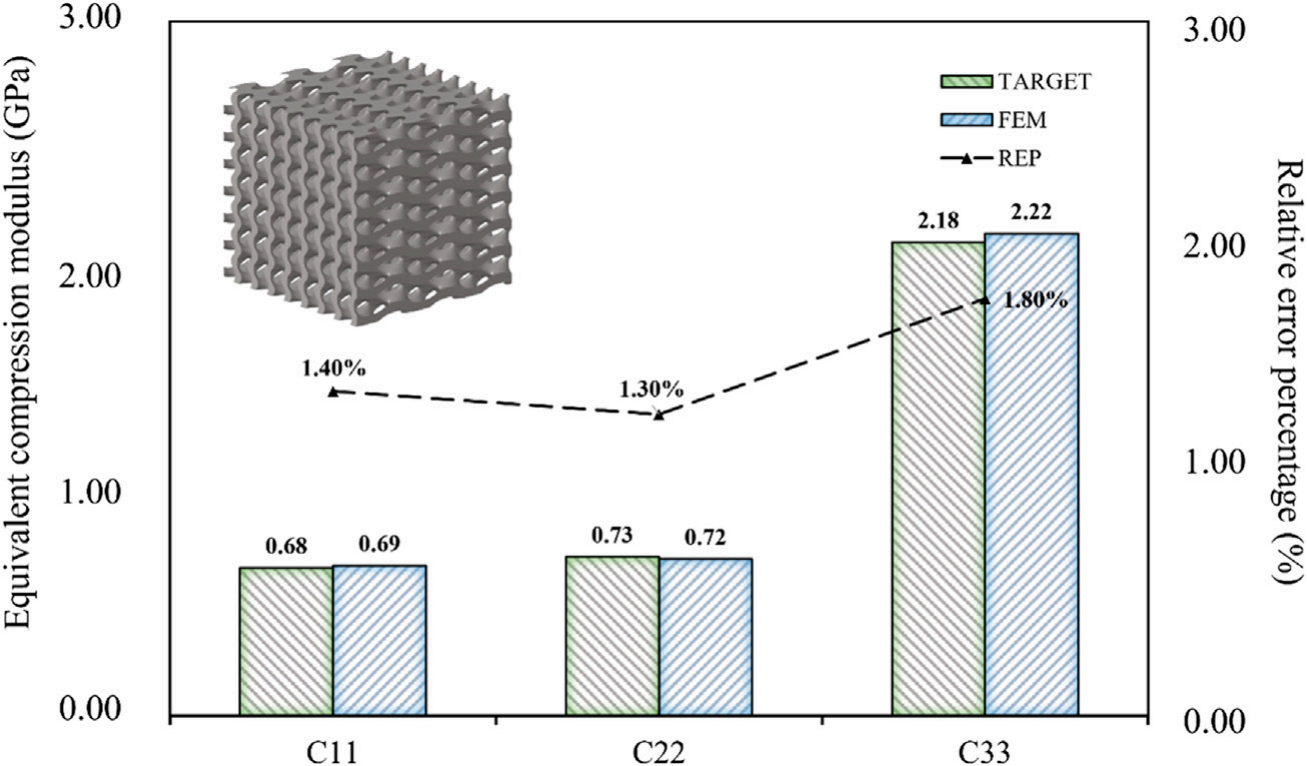
Error analysis of three design objectives of porous bone.

When the design targets were reduced to three, such as the compressive modulus in three directions, the relative error percentage became almost negligible (Figure 12). Therefore, achieving indiscriminate prediction of three or fewer design objectives within the current inverse design domain was possible. When the design target was only the three-dimensional compressive modulus, the maximum absolute error was limited to 0.04 GPa, and the maximum relative error percentage was limited to 2.00%. It can be concluded that the BPNN-RGA method has the higher design accuracy for the fewer design targets, as demonstrated by the decrease in relative error percentage.

### 3.3 Inverse design based on other porous bones

**FIGURE 13:**
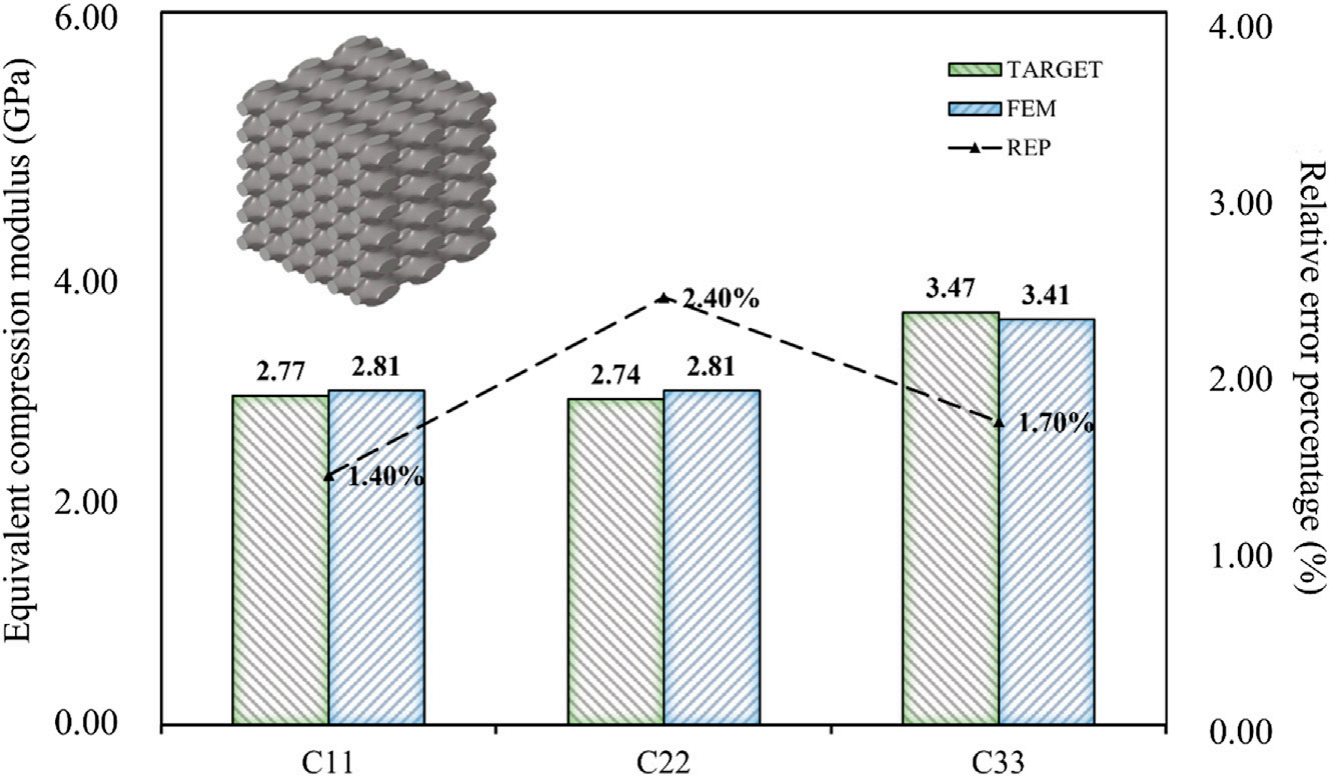
Error analysis of high porous bone based on existing literature.

The data presented in this article was obtained through the analysis of porous bone CT images. While its validity had been confirmed (Kalouche et al., 2010; Wang et al., 2016), it should be noted that it was served as an illustrative example. Due to the irregular arrangement of trabecular bone in porous bone, the modulus of different positions can vary greatly, and the ratio of modulus in each direction may be inconsistent. The three-dimensional compressive modulus from existing literature (Wu et al., 2018) was used as the design target to demonstrate the universality of the inverse design domain of the BPNN-RGA model (Figure 13).

As shown in Figure 13, the BPNN-RGA model still has good solutions for different compressive moduli and proportions. The corresponding TPMS structure was Primitive, the structural porosity was 52%, and the numbers in the *x*, *y*, and *z* directions were 3, 6, and 6, respectively. The maximum error of the structure is less than 3.00%. In addition to the inverse design of porous bone with a small modulus, low porosity bone with a relatively large modulus was also inverse designed. Design goals for low porosity bone from existing literature (Wang et al., 2016).

**FIGURE 14:**
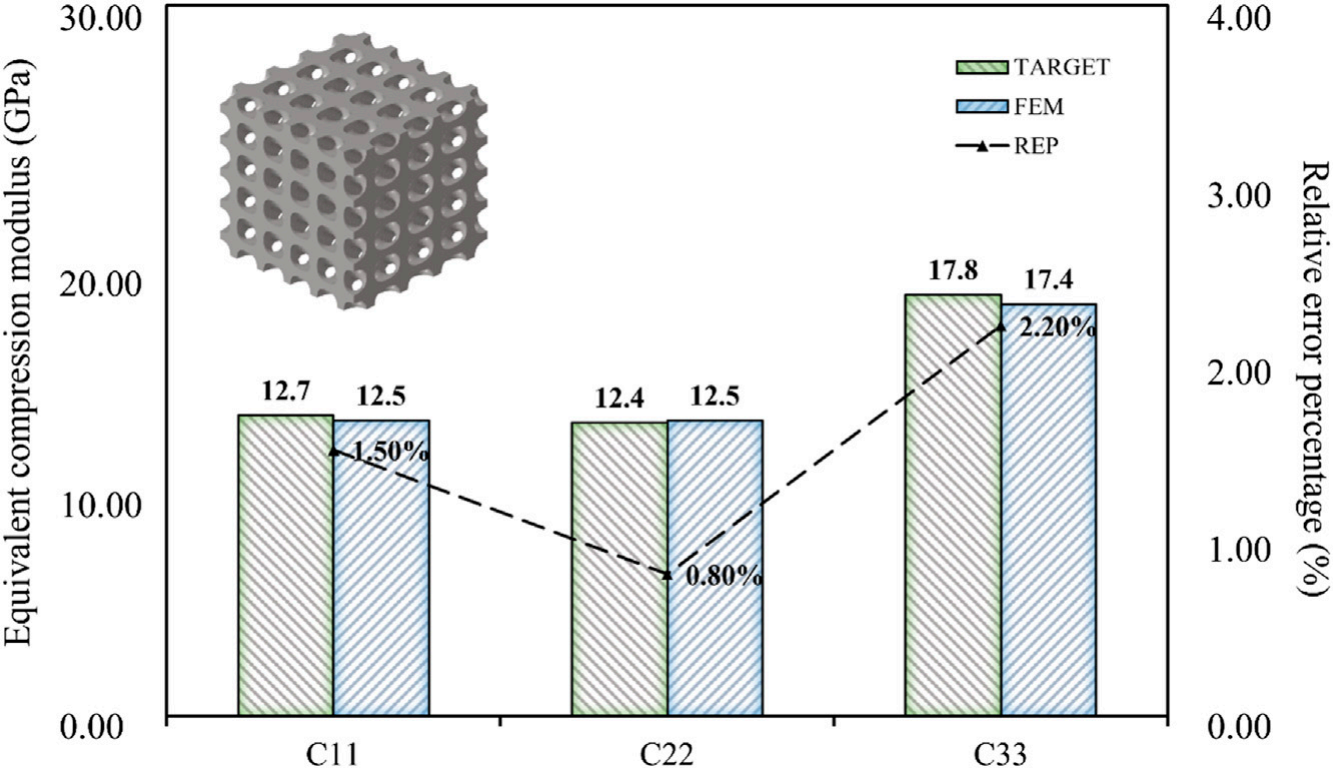
Error analysis of low porosity bone based on existing literature.

**FIGURE 15:**
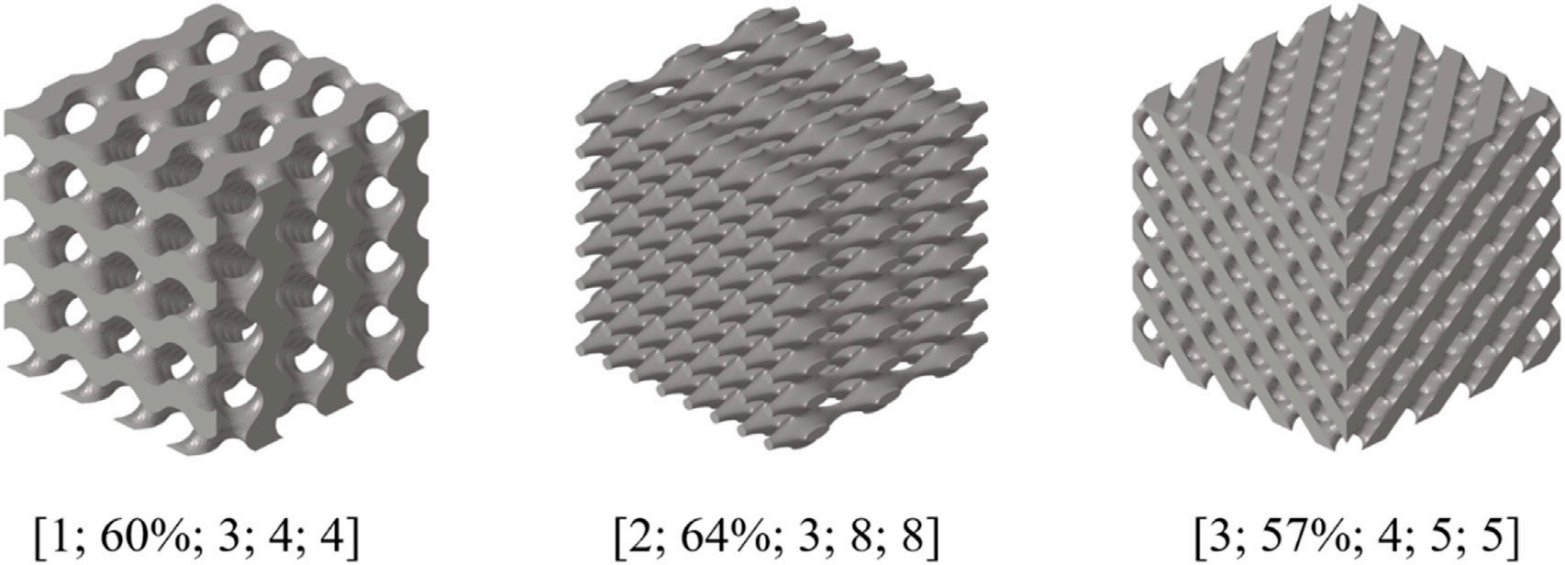
Several other structural diagrams that meet the design error.

As shown in Figure 14, the design error of BPNN-RGA for low porosity bone is still controlled within 3.00%. The corresponding TPMS structure was Octo, the structural porosity was 58%, and the numbers in the x, y, and z directions were 4, 5, and 5, respectively. In addition, the analysis of the historical output data of the RGA showed that when the control error was around 5.00%, several unit cell structures can satisfy the design requirements (Figure 15). These unit cells had different arrangement numbers in three directions and different porosity levels, providing more options for selecting the most appropriate porosity and structures for machining based on machining constraints.

## 4 Discussion

In the present study, a BPNN-RGA model was developed to design complex bone scaffolds, and a simulation error analysis was performed to verify the accuracy of the model. The present study indicates that complex bone scaffolds can be designed efficiently and accurately using the BPNN-RGA model.

In some ways, the design method based on neural networks was more efficient than other optimization methods. A comparative discussion with other bone scaffold design methods is given below: The randomization method based on computer aided design has been proven effective in simulating real bone through the randomization process (Mullen et al., 2010). However, this method requires many trials to achieve the expected performance. The design of unknown design targets could not be efficient. Although the BPNN-RGA model depends on data, when the data is accumulated enough, the neural network can find the internal relationship between the data to predict the unknown data accurately, which cannot be achieved by traditional randomization methods.

Except randomization, comparison with topology optimization methods is also a focus (Guest and Prevost, 2007). Utilized solid isotropic material with penalization based structural optimization to develop a topology optimization technique for finding a scaffold with pores in the shape of a Schwartz primitive structure. The topology optimization method was also used to match the stiffness matrix of the scaffold material to the stiffness matrix of anisotropic natural bone. Although topology optimization can realize the design of complex bones the designed structure may have gray units that affect the printing of the structure. It should be noted that although additive manufacturing (AM) can produce structures in any shape, the quality of the structures may vary greatly depending on the design and fabrication parameters (Wang et al., 2013). As the BPNN-RGA method is designed based on the TPMS structure, which has been widely used in bone scaffolds, there is no need to worry about manufacturing. The dimension of the design target is reduced using the parameters of the surface equation instead of the three-dimensional structures, which improves the design efficiency.

In addition, the selectivity of the design results are also the highlights of the BPNN-RGA model. When the design error was set to approximately 5.00%, we found various unit cells suitable for inverse design with the same design goals, which provided us with more options to select. The difference between the different types of TPMS structures, if they all met the design objectives, was the porosity. A successful implant must meet mechanical requirements that match the surrounding tissue to reduce stress shielding and prevent mechanical failure. However, except these considerations, it is also important to consider cell attachment and growth, as well as the transportation of nutrients and metabolic wastes for optimal biocompatibility (Langer and Vacanti, 1999; Hollister, 2009). Bone regeneration in porous implants *in vivo* involves the recruitment and penetration of cells from the surrounding bone tissue and vascularization (Karageorgiou and Kaplan, 2005). The porosity of a structure is linked to nutrient exchange and the size of its specific surface area. Therefore, when designing structures that meet specific requirements, we can make a decision based on the porosity of the structure (Story et al., 1998; Lewandrowski et al., 2000).

The limitation of the BPNN-RGA model is that the design field is limited to four TPMS structures. When there are more than six targets in the inverse design, some inaccuracies may occur due to the design field. To address this issue, one of the methods is to increase the variety of TPMS structures, and the other method is to increase the number of single-cell arrays. Furthermore, the efficiency of the BPNN-RGA model is reflected in its repeated use. The time cost of building the model may be higher if it is used only once. This is also a common feature of all machine learning models.

## 5 Conclusion

In this paper, a new inverse design method BPNN-RGA was proposed to inversely design anisotropic bone scaffolds. In this method, anisotropy was introduced into the arrangement of bone scaffolds based on traditional TPMS structures, and the bone stiffness matrix calculated from CT images was used as the inverse design target. The results of the FE calculation were used in neural network training to find the mapping relationship between the structural parameters and the elastic modulus. RGA was used in inverse design to find the structure meeting the target modulus. Multiple bone data were used to verify the universality and accuracy of the BPNN-RGA method. The results showed that the average error was less than 3.00%, when the design targets was three mechanical performance parameters and about 5.00% when the design targets was six. Compared with the traditional optimization method, the proposed BPNN-RGA model achieves high design efficiency. Moreover, the results of the design have the characteristics of stability and selectivity.

## Data Availability

Original research data in the study are included in the article, further inquiries can be directed to the corresponding author.
